# Biogeography of the two major arbovirus mosquito vectors, *Aedes aegypti *and *Aedes albopictus *(Diptera, Culicidae), in Madagascar

**DOI:** 10.1186/1756-3305-5-56

**Published:** 2012-03-20

**Authors:** Fara Nantenaina Raharimalala, Lala Harivelo Ravaomanarivo, Pierre Ravelonandro, Lala Sahondra Rafarasoa, Karima Zouache, Van Tran-Van, Laurence Mousson, Anna-Bella Failloux, Eléonore Hellard, Claire Valiente Moro, Bakoly Olga Ralisoa, Patrick Mavingui

**Affiliations:** 1 UMR CNRS 5557 Ecologie Microbienne, Université Lyon 1, 43 boulevard du 11 Novembre 1918, Villeurbanne cedex 69622, France; 2 Département d'Entomologie, de la Facultés des Sciences d'Antananarivo, Antananarivo, Madagascar; 3 Centre National de Recherche sur l'Environnement, Antananarivo, Madagascar; 4 Institut Pasteur, Département de Virologie, Laboratoire Arbovirus et Insectes Vecteurs, Paris, France; 5 CNRS; UMR 5558, Laboratoire de Biométrie et Biologie Evolutive, Université Lyon 1, Université de Lyon, Lyon, Villeurbanne F-69622, France; 6 Laboratoire Arbovirus et Insectes Vecteurs, Institut Pasteur, Paris, France

## Abstract

**Background:**

In the past ten years, the Indian Ocean region has been the theatre of severe epidemics of chikungunya and dengue. These outbreaks coincided with a high increase in populations of *Aedes albopictus *that outcompete its sister taxon *Aedes aegypti *in most islands sampled. The objective of this work was to update the entomological survey of the two *Aedes *species in the island of Madagascar which has to face these arboviroses.

**Methods:**

The sampling of *Aedes *mosquitoes was conducted during two years, from October 2007 to October 2009, in fifteen localities from eight regions of contrasting climates. Captured adults were identified immediately whereas immature stages were bred until adult stage for determination. Phylogenetic analysis was performed using two mtDNA genes, *COI *and *ND5 *and trees were constructed by the maximum likelihood (ML) method with the gene time reversible (GTR) model. Experimental infections with the chikungunya virus strain 06.21 at a titer of 10^7.5 ^pfu/mL were performed to evaluate the vector competence of field-collected mosquitoes. Disseminated infection rates were measured fourteen days after infection by immunofluorescence assay performed on head squashes.

**Results:**

The species *Aedes aegypti *was detected in only six sites in native forests and natural reserves. In contrast, the species *Aedes albopictus *was found in 13 out of the 15 sites sampled. Breeding sites were mostly found in man-made environments such as discarded containers, used tires, abandoned buckets, coconuts, and bamboo cuts. Linear regression models showed that the abundance of *Ae. albopictus *was significantly influenced by the sampling region (F = 62.00, p < 2.2 × 10^-16^) and period (F = 36.22, p = 2.548 × 10^-13^), that are associated with ecological and climate variations. Phylogenetic analysis of the invasive *Ae. albopictus *distinguished haplotypes from South Asia and South America from those of Madagascar, but the markers used were not discriminant enough to discern Malagasy populations. The experimental oral infection method showed that six *Ae. albopictus *populations exhibited high dissemination infection rates for chikungunya virus ranging from 98 to 100%.

**Conclusion:**

In Madagascar, *Ae. albopictus *has extended its geographical distribution whereas, *Ae. aegypti *has become rare, contrasting with what was previously observed. Changes are predominantly driven by human activities and the rainfall regime that provide suitable breeding sites for the highly anthropophilic mosquito *Ae. albopictus*. Moreover, these populations were found to be highly susceptible to chikungunya virus. In the light of this study, *Ae. albopictus *may have been involved in the recent outbreaks of chikungunya and dengue epidemics in Madagascar, and consequently, control measures should be promoted to limit its current expansion.

## Background

Among the 29 mosquito species of the genus *Aedes *reported in Madagascar, thirteen are endemic [1]. Since the reports in the 1980s [1,2], no extensive survey has been conducted on the geographical distribution of the *Aedes *genus in Madagascar. As *Aedes *species are major vectors of arboviral diseases, the control of these mosquitoes is needed to prevent or limit the epidemic risks. Recently, the species *Aedes aegypti *and *Aedes albopictus *have been involved in arbovirus outbreaks worldwide [3-5]. During the last ten years, the Indian Ocean Islands have witnessed severe epidemics of arboviruses, notably chikungunya (CHIK) and dengue (DEN). In contrast to the 1950s when *Ae. aegypti *was involved as the main vector [6,7], the species *Ae. albopictus *has been identified as the primary vector of most recent outbreaks in the Indian Ocean [8-12].

In Madagascar, outbreaks of DEN and CHIK fevers emerged in the east coast of Toamasina on January 2006 [11]. Since then, several cases of CHIK and DEN were reported in different regions of Madagascar, including Antalaha (north-east coast), Antsiranana (north coast), Mahajanga (north-west coast) and Toamasina (east coast) [13], (http://www.invs.sante.fr). During these epidemics, the species *Ae. albopictus *was identified as the main vector [5,11,14]. These data are in line with what is known on the current worldwide expansion of *Ae. albopictus*, which outcompetes its sister taxon *Ae. aegypti *[15-18].

The goal of this study was to survey populations of *Ae. aegypti *and *Ae. albopictus *of Madagascar and to explore whether their geographical distribution has evolved. Entomological investigations were conducted in eight regions where various sites were visited. The choice of the areas to sample was based on at least one of the following elements (i) existence of previous records of the genus *Aedes *spp, (ii) contrasted ecoclimatic characteristics, and (iii) suspected or confirmed human cases of CHIK and/or DEN during recent outbreaks in the region [11,13,19]. Collected adults and immature stages were genetically characterized by haplotyping, and susceptibility to arboviruses was measured in a biosafety laboratory level 3.

## Methods

### Geographic location and characteristics of study areas

**Figure 1:**
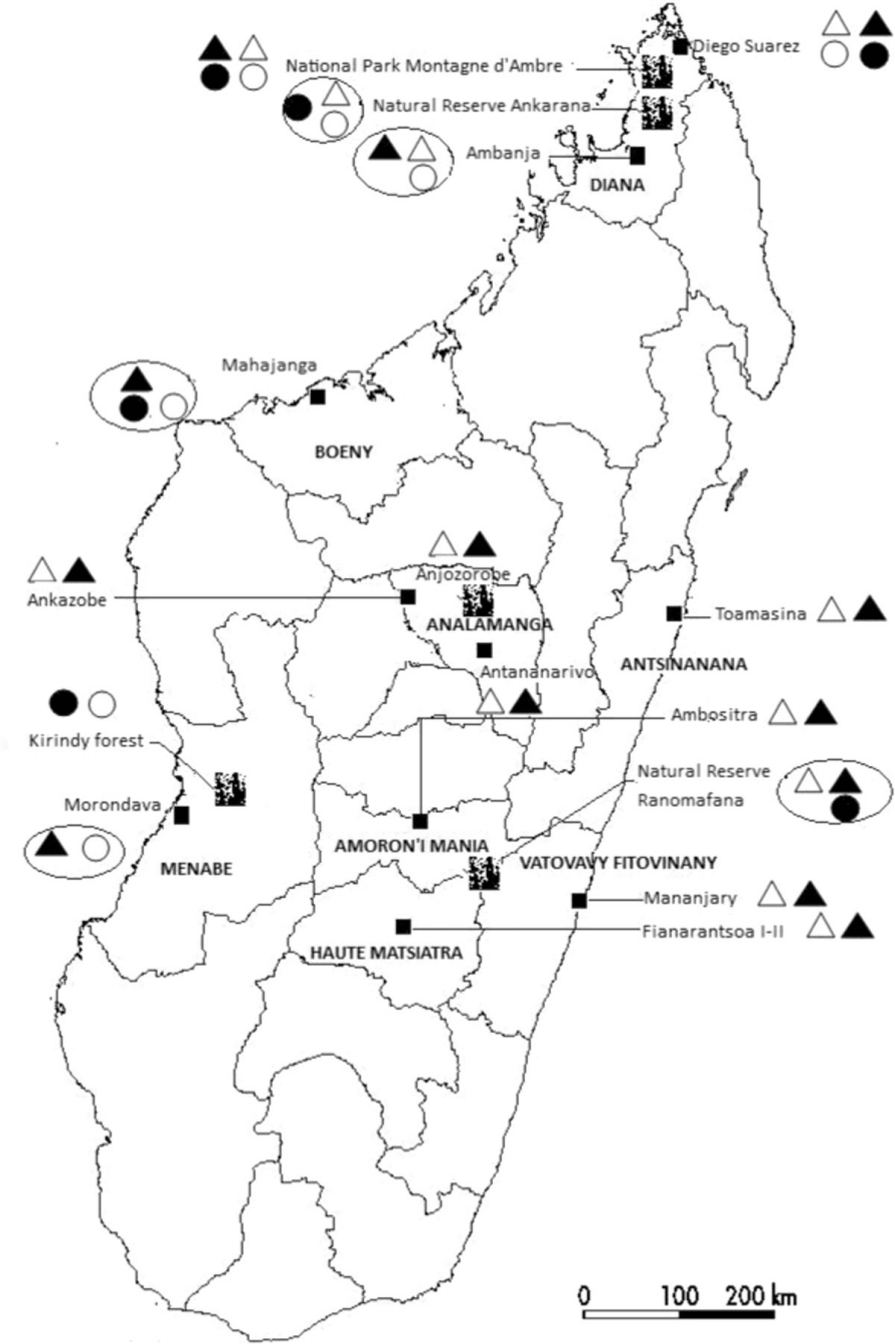
**Distribution of *Aedes *mosquitoes in eight regions of Madagascar**. **Empty triangle *Aedes albopictus *and empty circle *Aedes aegypti *in the 1980s (Ravaonjanahary, 1978; Fontenille et al., 1989)**. Filled triangle *Aedes albopictus *and filled circle *Aedes aegypti *at the end of 2009 (this study). Encircled symbols highlighted changes in species distribution. Black filled square, town. Mosaic square, forest areas.

**Figure 2:**
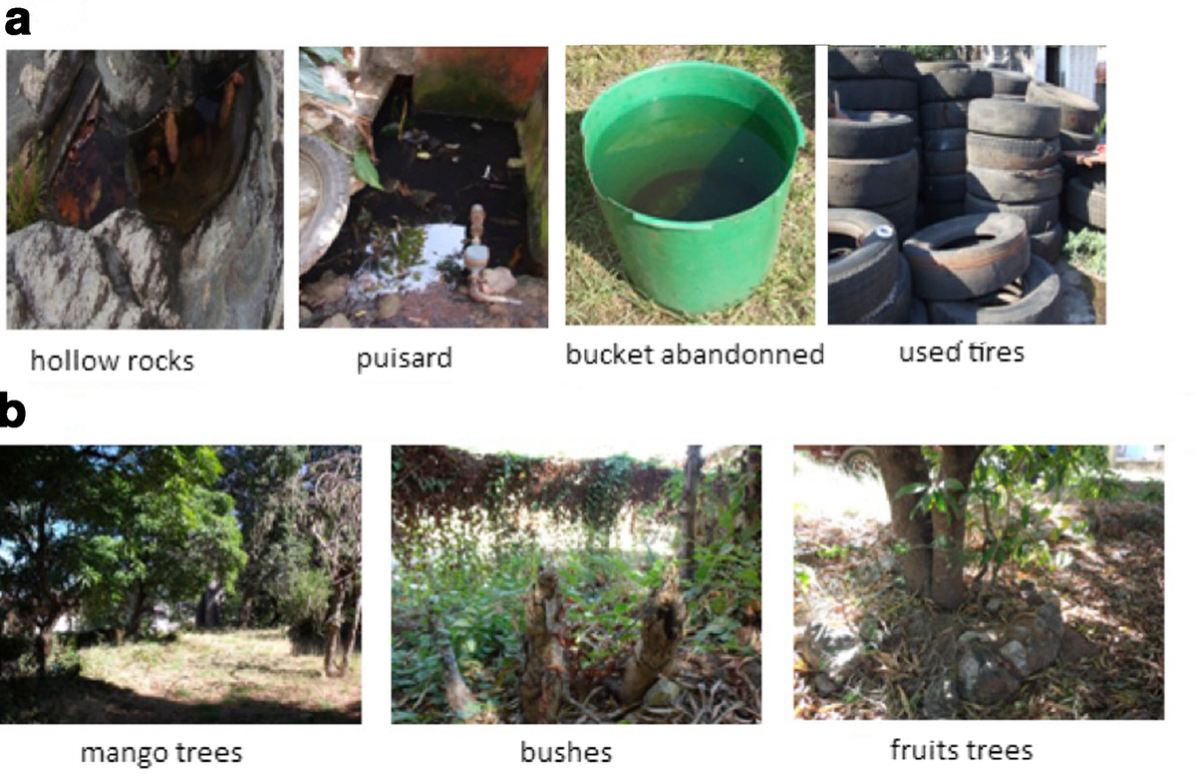
**View of mosquito breeding sites**. **A: sites of immature stages; B: sites of adults**.

*Ae. albopictus *and *Ae. aegypti *were sampled in cities, villages, natural reserves and forests in eight regions of Madagascar (Figure 1). The characteristics of the sampled areas are summarized in Table 1 and Figure 2. The Analamanga region is located in the highlands of Madagascar at an altitude of 1200-1433 m, with a tropical climate. In this area, two seasons are observed: a hot (21°C on average) and rainy period (November to April with about 200 mm precipitation in a month), and a relatively dry cool season for the rest of the year (10°C on average and rainfall not exceeding 20 mm precipitation in a month). The Zoological and Botanic Park of Tsimbazaza is located in the Center of Antananarivo at 1200 m altitude. As Analamanga region, Amoron'i Mania region (Ambositra) and Haute Matsiatra region (Fianarantsoa) are also in the highlands of Madagascar and have similar features. Ranomafana forest is situated in a region between Amoron'i Mania and Vatovavy Fitovinany in the western side of Madagascar. The climate is tropical, humid and rainy (with an annual rainfall of 2600 mm) and the temperature varies from 14 to 20°C. The Antsinanana region (Toamasina, Foulpointe) is in the eastern plains of the island. The climate is hot and wet and rains occur all the year with the mean annual rainfall of about 3200 mm and a relative humidity around 87%.

**Table 1:** Ecological characteristics of sampling areas

Areas	Regions	Sites	Adult breeding sites	Larval habitats
Highlands	Analamanga	Antananarivo city	Bamboo	Bamboo hedge, discarded containers
		Anjozorobe forest	Bamboo, bushes	Bamboo hedge
		Ankazobe village	Bushes	Bamboo hedge, used tires
	Amoron'i Mania	Ambositra city	Fruit trees, bamboo, bushes	Bamboo hedge, used tires
		Ranomafana village, forest	Bushes	Discarded containers, hollow roks
	Haute Matsiatra	Fianarantsoa I-II city	Mango trees, bushes	Discarded containers, used tires
Eastern Plains	Antsinanana	Toamasina city in coast	Bamboo hedge, bushes	Abandoned buckets, used tires, drum, coconut
Western Plains	Boeny	Mahajanga city in coast	Mango trees, bushes	Discarded containers, used tires
	Menabe	Morondava city in coast	Mango trees, bushes	Abandoned buckets, used tires, drum, coconut
		Kirindy forest	Forest	Hollow rocks, tree holes
Southeast Coast	Vatovavy Fitovinany	Mananjary city in coast	Fruit trees, bushes	Bamboo hedge, discarded containers, coconut
North Plains - North West	Diana	Ambanja city	Bushes	Abandoned buckets, used tires, drum, coconut
		Ankarana forest	Forest	Hollow rocks, tree holes
		Montagne d'Ambre forest	Forest	Hollow rocks, tree holes
		Diego Suarez city in coast	Bushes, fruit trees	Abandoned buckets, used tires, drum, coconut

The Boeny region (Mahajanga) is located in the western plains. It has an equatorial climate with an arid and warm summer (mean temperature of 27°C) with moderate rainfall (the mean precipitation is about 400 mm per year). The Menabe region (Morondava, Kirindy forest) is also situated in the western plains. It has a similar climate to the Boeny region. However, the temperature can reach more than 40°C during the rainy season (late December until the end of March). Rainfall is not constant during this period but spaced with dry periods.

The Vatovavy-Fitovinany region (Mananjary) is in the southwest coast of Madagascar and has a tropical climate along the coast, is temperate inland and arid in the south. The Diana region is situated in the north-western plains of Madagascar, where the sampling sites of Diego-Suarez and Ambanja cities are located, as well as the reserved forests of Ankarana and Montagne d'Ambre. The climate is equatorial dry and this area is situated between 4 m altitude (Ankarana) and 1400 m (Montagne d'Ambre). It is characterized by an alternation of wet and dry seasons (May to November) and a hot and humid season (December to April). The average temperature is 27°C and rainfall varies from 985 to 2171 mm per year.

### Collection and processing of mosquitoes

Adults and immature stages of mosquitoes were collected monthly from October 2007 to October 2009 in 8 regions corresponding to a total of 15 different sites (Figure 2). In each site, collections were performed daily over a period of one week by visiting three to four spots previously identified as hosting adult breeding sites and larval habitats of *Aedes *(Table 1). The same spots were visited, unless changes occurred due to human activities, thus obliging capturers to search for other potential new breeding sites and habitats in the area. As *Aedes albopictus *and *Aedes aegypti *are diurnal mosquitoes, adults were collected during the two peaks of biting activities, i.e. 7:00 to 10:30 am and 15:30 to 18:00 pm. Two methods were used to catch adult mosquitoes. The first method consisted of the use of butterfly nets that allows collection of both females and males flying near the grass. This was done by applying ten rounds of netting near the grassland and underneath bushes, bamboo and fruit trees. Then, capturers moved to another site. The second method was performed using oral aspirators to capture host-seeking females landing on the legs of human volunteer-baits who gave their agreement. Malagasy administrative authorities, particularly Madagascar National Parks (formely ANGAP), were informed and approved mosquito collections which were conducted in conformity to the declaration of Helsinky on ethical principles for medical research involving human subjects. To avoid biting, three capturers performed collection at one time. Exposed legs of one volunteer were under monitoring of two non-exposed capturers who catch mosquitoes immediately after landing. When trapping in an area, capturers spent 15 to 30 min at one spot then moved to another. Each spot was visited twice during each peak of biting activity. Collected adult mosquitoes were stored in cups covered with netting. *Aedes *spp. specimens were identified morphologically [1], then males and females were separately desiccated, in silicagel. In order to keep some individuals alive during transfer to the laboratory, impregnated cotton with 6% sucrose solution was placed at their disposal. Larval habitats consisted of artificial containers and natural sites containing standing water (Table 1). When larvae and pupae were present, they were collected using a dipper and transferred to a plastic bottle using a wide-mouthed pipette. Collected specimens were brought back to the laboratory where they were reared until adult stage and then identified. Usually, larvae and pupae were sampled at the same time as adults. For each species, the number of individuals, date of capture and location site were recorded.

### Mosquito infection and virus dissemination rate measurement

Wild-caught females were blood-fed on chickens and allowed to lay their eggs on absorbent paper in insectaries under standard conditions (28°C ± 2°C, 80% ± 12% humidity, photoperiod: 12 h/12 h) in Madagascar. Batches of eggs were brought to biosafety insectaries in the Pasteur Institute in Paris using a safety transport procedure. After hatching, larvae were reared to adults in standard conditions as described [20].

To infect mosquitoes, two independent experiments were carried out at an interval of three months with the same batches of eggs. The strain CHIKV (E1-226 V), which has an A- > V amino acid substitution at position 226 in the E1 glycoprotein, was used [8]. Virus stock and mosquito infection were performed as described [9,20]. Briefly, 1 mL of viral suspension was added to 2 mL of washed rabbit erythrocytes supplemented with ATP (5 × 10^-3 ^M) as a phagostimulant. The resulting infectious blood at a titer of 10^7.5 ^PFU/mL was transferred to a glass feeder at 37°C on top of the mesh covering a plastic box containing female mosquitoes from each of the six populations that were previously starved for 24 h. After 15 min of oral feeding, mosquitoes were sorted on ice. Fully engorged females were transferred to cardboard containers then fed with 10% sucrose at 28°C for 14 days. After the incubation period, disseminated infection rates of CHIKV were determined (using surviving live mosquitoes), by immunofluorescence assay (IFA) on head squashes as described [21]. In brief, mosquito heads were placed between two glass slides and squashed by hand pressure. Squashed tissues were fixed by immersing in acetone for 20 min at 20°C, then incubated with a first antibody, anti-CHIKV diluted in PBS 1X (1:200); the antibody was obtained from a mouse ascite. After incubation for 30 min at 37°C, slides were washed three times in PBS 1X and incubated with an anti-mouse conjugate diluted in PBS 1X (1:80) and supplemented with Evan blue. Slides were observed under an epifluorescence microscope (Leitz, Larbolux K). In positive samples, the head tissue appeared in green whereas blue-colored tissue is negative. Therefore, the disseminated infection rate corresponds to the ratio between positive heads to the total number of surviving individuals examined.

### Nucleic acid extraction, PCR amplification and sequencing

To extract genomic DNA, five males and five females from each site were washed three times with sterilized water, then surface-disinfected with ethanol 80% for 5 min. After five washes with sterilized water, each individual mosquito was homogenized and the genomic DNA extracted using the procedure previously described [22].

For PCR, amplification was performed using a T Gradient Thermocycler (Biometra, France) with the primers targeting two mtDNA gene fragments: a 597-bp fragment of *COI *(cytochrome-oxydase subunit 1) and a 450-bp fragment of *ND5 *(NADH dehydrogenase subunit 5). The two sets of primers used were: for *COI*, CI-J-1632 (5'-TGATCAAATTTATAAT-3') and CI-N-2191 (5'-GGTAAAATTAAAATATAAACTTC-3') [23]; and, for *ND5*, ND5FOR (5'-TCCTTAGAATAAAATCCCGC-3') and ND5REV (5'-GTTTCTGCTTTAGTT-CATTCTTC-3') [24]. Reactions were made in 50 μl volume containing 90 ng of DNA template, 1× PCR buffer, 50 mM MgCl2, 10 μM of each primer, 2 mM of dNTP mix (INVITROGEN, France), 0.4 U of *Taq *DNA polymerase (INVITROGEN, France). The temperature profile for *ND5 *consisted of an initial denaturation at 98°C for 2 min, followed by 5 cycles of 95°C for 30 s, 45°C for 30 s, 72°C for 45 s, then 25 cycles of 95°C for 30 s, 46°C for 45 s, 72°C for 45 s, and a final extension at 72°C for 5 min. For *COI *the amplification program consisted of an initial denaturation at 95°C for 5 min, followed by 35 cycles of 97°C for 30 s, 40°C for 45 s, and 72°C for 1 min, and a final extension at 72°C for 5 min [25]. An aliquot of 10 μl of each PCR product was subjected to electrophoresis on a 1% agarose gel at 50 V for 30 min, then stained with ethidium bromide and photographed with Gel Doc 2000 system (BioRad, USA). When bands with expected size were visualized, the remaining PCR products (approximately 40 μl) were sent to sequencing at BIOFIDAL-DTAMB (FR BioEnvironment and Health, Lyon, France).

### Phylogenetic analysis

The *COI *and *ND5 *gene fragments were sequenced in both strands and deposited in Genbank with accession numbers JN406654-JN406797, JN406804-JN406809, JN406822-JN406832, JN406839-JN406844. Sequences were then aligned with those of mosquitoes available in databases by using BioEdit and Multialn softwares. The two gene sequences were concatenated to improve the reliability of the phylogenetic analysis. Phylogenetic analysis was carried out by using the Seaview software (http://pbil.univ-lyon1.fr/software/seaview.html) based on maximun likelihood (ML), maximum parsimony (MP) and neighbor-joining (NJ) methods. Trees were then constructed with the general time reversible (GTR) model, and branch supports were estimated by bootstrapping with 1000 replicates.

### Statistical analysis

The effects of the sampling region, year, period, main habitat type and presence of one mosquito species to the abundance of the other species were investigated using linear regression models. The variable "period" corresponded to subdivision in a three-months sampling period per year, which can be linked roughly to a more rainy season from October to March and a more dry season from April to September. The variable "area" was categorized as highlands, eastern plains, western plains, southeast coast, north plains and north east (Table 1). All possible models (with all subsets of variable numbers and combinations) were generated. A square transformation was applied to ensure the normality of the residuals. Due to the sample size, only the region*year interaction was tested. Area and region were never put together in a model because they were correlated. The most appropriate model was selected using the Akaike Information Criterion adjusted for small sample size (AICc, [26]). The models were ranked according to the smallest AICc differences (denoted ΔAICc) between the focal model and the lowest AICc model. When ΔAICc was larger than 2, the model with the smallest AICc was selected. On the contrary, when ΔAICc was smaller than 2 and models nested, the most parsimonious model was kept. When models were not nested but AICc smaller than 2, both models were kept.

## Results

### Distribution of *Aedes *in the sampled regions of Madagascar

Immature stages and adults of *Ae. albopictus *predominated in most sampled sites (13 out of 15). During the 27 months of sampling, a total of 12,639 adults (9891 females and 2748 males) and 7891 immature specimens of *Ae. albopictus *were collected (Table 2). *Aedes albopictus *was found in diverse ecological areas, from highland to the coast and under contrasting climates from arid to humid. Breeding sites and habitats included natural and human-made environments. In contrast, the species *Ae. aegypti *was found in very limited numbers as imago (269 females and 46 males) and was mostly confined to sylvatic zones (Ranomafana, Kirindy, Ankarana and Montagne d'Ambre forest), with only a few specimens also captured in Diego Suarez city (Table 3). In some areas, the current distribution of the two species was significantly different from what was recorded in the 1980s (Figure 1). For instance, in Ambanja town (Diana region), the two species were reported to be sympatric, but during our investigation, only *Ae. albopictus *was found. This coincided with the extension of the urbanization zone and an increase of man-made breeding sites. Interestingly, a converse pattern was observed in neighbouring villages of Ankarana where the sympatry was substituted by the allopatry of *Ae. aegypti*. In the same line, *Ae. aegypti *has recently colonized the natural reserve of Ranomafana, whereas *Ae. albopictus *has extensively invaded the cities of Mahajanga (Boeny region) and Morondava (Menabe region).

**Table 2:** Abundance of *Aedes albopictus *captured in sampling regions.

Species	Aedes albopictus numbers*										
MonthsRegions	Oct-Dec(2007)	Jan-Mar(2008)	Avr-Jun(2008)	Jul-Sept(2008)	Oct-Dec(2008)	Total2007-2008	Jan-Mar(2009)	Avr-Jun(2009)	Jul-Sept(2009)	Oct-Dec(2009)	Total2009
Analamanga	595	493	328	287	581	**2284**	553	242	237	511	**1543**
Amoron'i Mania	64	72	12	15	73	**236**	68	32	24	103	**226**
Haute Matsiatra	37	31	7	9	29	**113**	67	48	21	43	**179**
Antsinanana	1021	923	116	178	899	**3137**	1549	256	197	1762	**3764**
Boeny	279	213	106	84	249	**931**	253	142	137	211	**743**
Menabe	122	88	39	21	171	**441**	101	91	65	180	**437**
Vatovavy Fitovinany	687	586	268	216	682	**2439**	958	269	287	827	**2341**
Diana	213	175	129	108	247	**872**	259	117	214	254	**844**
**Total**						**10453**					**10077**

Data are grouped per three-months sampling period. *Aedes albopictus numbers corresponded to the total of wild adults collected from the field and immature stages collected from the field and emerged in the laboratory (both sexes).

**Table 3:** Abundance of *Aedes aegypti *in sampling regions.

Species	Aedes aegypti numbers*										
MonthsRegions	Oct-Dec(2007)	Jan-Mar(2008)	Avr-Jun(2008)	Jul-Sept(2008)	Oct-Dec(2008)	Total2007-2008	Jan-Mar(2009)	Avr-Jun(2009)	Jul-Sept(2009)	Oct-Dec(2009)	Total2009
Analamanga	0	0	0	0	0	**0**	0	0	0	0	**0**
Amoron'i Mania	0	0	0	0	0	**0**	0	0	0	0	**0**
Haute Matsiatra	0	0	0	0	0	**0**	0	0	0	0	**0**
Antsinana	0	0	0	0	0	**0**	0	0	0	0	**0**
Boeny	10	6	4	0	6	**26**	15	0	0	8	**23**
Menabe	20	11	3	2	17	**53**	22	2	5	13	**42**
Vatovavy FitovinanY	0	0	0	0	0	**0**	0	0	0	0	**0**
Diana	32	24	7	9	27	**99**	31	7	6	28	**72**
**Total**						**178**					**137**

Data are grouped per three-months sampling period. *Aedes aegypti numbers corresponded to the total of wild adults collected from the field, no immature stages were recorded.

As *Ae. albopictus *has expanded since the 1980's in Madagascar and now predominated in most sampled sites, further analyses were focused on that species. Statistical analysis showed that the abundance of *Ae. albopictus *was best described by a linear regression model including a regional effect (F = 62.00, df = 7.58, p < 2.2 × 10^-16^) and a period effect (F = 36.22, df = 3.58, p = 2.548 × 10^-13^) (Table 4). There was an increasing abundance in sequential order Haute Matsiatra, Amaoron'I Mania, Menabe, Boeni, Diana, Analamanga, Vatovavy Fitovinany, and Antsinanana, whereas less *Ae. albopictus *was captured from July to September and April to June, that corresponded to more dry season, than from January to March and October to December (Table 5). There was no significant variation of *Ae*. *albopictus *from year to year during the study period (Table 6).

**Table 4:** Best linear models for *Aedes albopictus *abundance according to the AICc.

Model	n	k	AICc	ΔAICc
Sqrt(AA) ~ Site + Period + Year	69	13	339.80	
**Sqrt(AA) ~ Site + Period**	**69**	**12**	**340.82**	**1.02**
Sqrt(AA) ~ Site + Period + Year + AE	69	14	342.16	2.35
Sqrt(AA) ~ Site + Period + AE	69	13	342.85	3.05
Sqrt(AA) ~ Site*Year + Period	69	20	347.82	8.02

The retained model is in bold. AA stands for *Ae. albopictus *(abundance), AE for *Ae. aegypti *(presence/absence), n for sample size (three outliers were removed) and k for the number of parameters. Only the five best models are shown but all possible models (i.e., with all variable numbers and combinations) were built.

**Table 5:** Selected linear regression model to describe *Aedes albopictus *abundance: parameters.

Variable	**β**^**a**^	**SE**^**b**^	95% CI^c ^(β)	
			min	max
Intercept	3.27	1.01	1.29	5.25
Site ANA	12.29	1.19	9.96	14.61
Site ANT	17.42	1.33	14.81	20.02
Site BOE	6.59	1.19	4.26	8.91
Site DIA	6.83	1.19	4.50	9.15
Site HAU	-1.38	1.19	-3.70	0.95
Site MEN	2.68	1.19	0.35	5.00
Site VAT	15.48	1.19	13.16	17.81
Period JM	5.97	0.91	4.20	7.75
Period JS	0.05	0.91	-1.72	1.83
Period OD	6.62	0.82	5,01	8.23

^a ^Parameter estimation; ^b ^Standard error; ^c ^Confidence interval.

JM, January to March. JS, July to September. OD, October to December

**Table 6:** Models to describe *Aedes albopictus *abundance with simple effects.

model	n	k	F	df (model; error)	P	AICc
sqrt(AA) ~ Region	72	9	11.31	(7; 64)	3.11 × 10^-9^	1006.63
sqrt(AA) ~ Area	72	6	14.35	(4; 67)	1.63 × 10^-8^	1012.43
sqrt(AA) ~ Period	72	5	4.23	(3; 68)	0.008	1042.28
sqrt(AA) ~ AE	72	3	4.85	(1; 70)	0.03	1045.23
sqrt(AA) ~ Year	72	3	0.01	(1; 70)	0.91	1050.04

AA stands for *Ae. albopictus *(abundance), AE for *Ae. aegypti *(presence/absence), n for sample size (three outliers were removed) and k for the number of parameters.

### Phylogenetic analysis of *Aedes albopictus*

**Figure 3:**
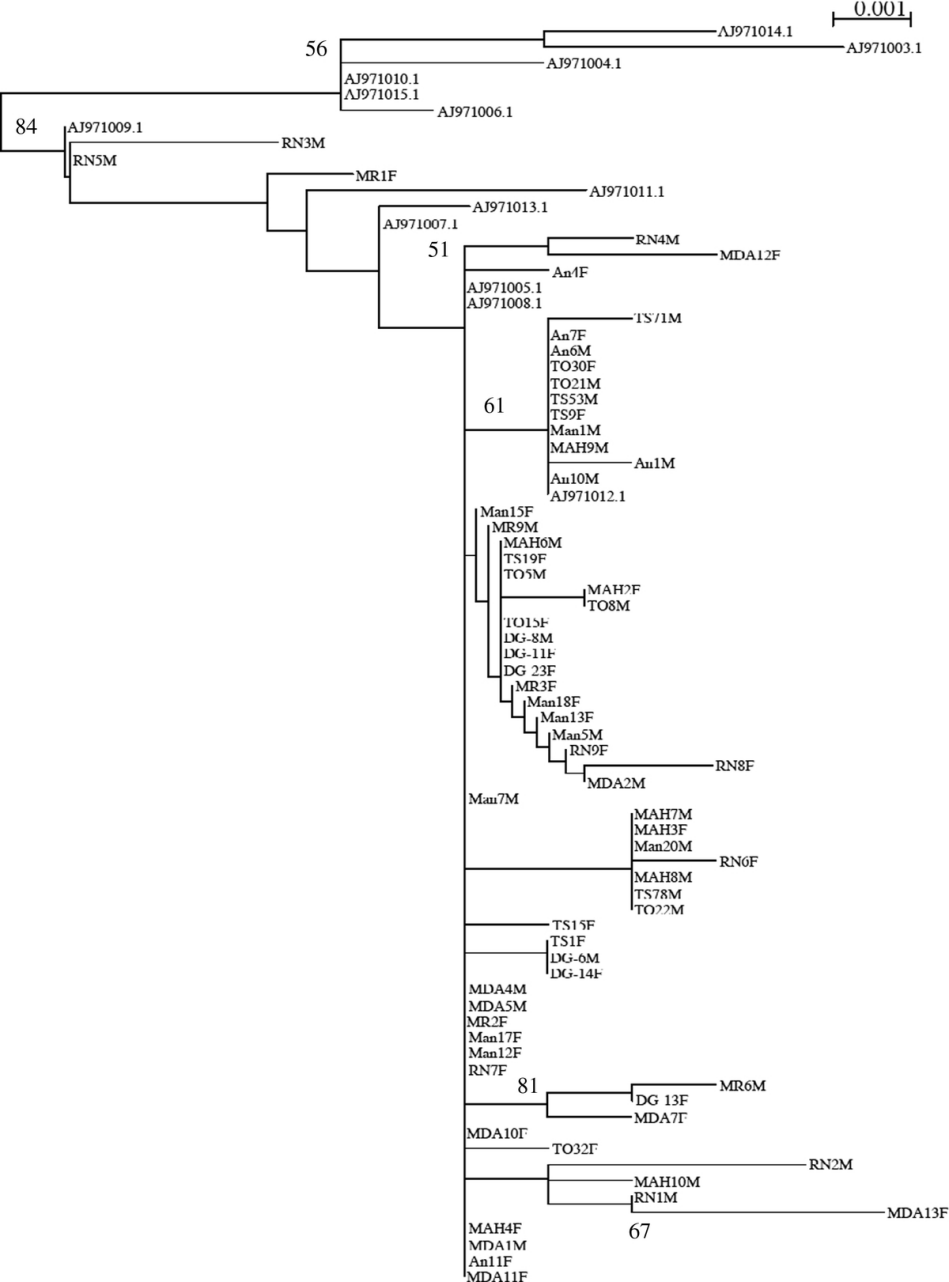
**Phylogeny inference from concatenated *COI *and *ND5 *genes of *Aedes albopictus *by the maximum likelihood (ML) method based on the general time reversible (GTR) model**. Percentage bootstrap supports (1000 replicates) superior to 50% are given at each node. Branch lengths represent estimated substitutions per site. Code sequence is as follows: An (Ankazobe), DG (Diego-Suarez), MAH (Mahajanga), Man (Mananjary), MDA (Montagne d'Ambre), MR (Morondava), RN (La Réunion), TO (Toamasina), TS (Tsimbazaza). Sequences retrieved from Genbank belonged to specimens from Brasil (AJ971003.1 and AJ971014.1), Vietnam (AJ971004.1 and AJ971010.1), Thailand (AJ971015.1), Cambodia (AJ971006.1), France (AJ971009.1 and AJ971008.1), Hawai (AJ971011.1), USA (AJ971005.1), La Reunion (AJ971013.1 and AJ971012.1), and Madagascar (AJ971007.1).

The sequences of two genes (449 bp for *ND5 *and 597 bp for *COI*) were obtained from 80 *Ae*. *albopictus *(5 females and 5 males for each site) and were aligned with similar sequences retrieved from the Genbank database (Figure 3). The alignment revealed substitutions consisted of both transitions and transversions (not shown). The tree built from concatenated sequences (1046 bp) identified two well-defined groups (Figure 3), with a strong support bootstrap value (84%). One group consisted of specimens from South America (Brazil) and South-Asia (Cambodia, Thailand, Vietnam), and the other clustered all the sequences from Madagascar together with those of the Indian Ocean (La Reunion), North America (USA and Hawaii) and Europe (France). In Madagascar, the data of *COI *and *ND5 *separately (not shown) or concatenated (Figure 3) did not cluster *Ae. albopictus *specimens according to neither geographical location nor urban, suburban and sylvatic habitats. For example, individuals from Montagne d'Ambre forest (North West) were not distinguishable from those collected in Toamasina city (Eastern plains) or Ankazobe village (Highlands). As females have a dispersal behaviour driven by the search for oviposition sites, which are in turn influenced by natural or domestic environments, a phylogenetic analysis was performed according to sex. However, similar tree topologies were obtained (not shown). Sequences were intermixed between individuals sampled in contrasted ecological niches (bamboos, bushes, fruit trees, containers, tires etc.).

### Vector competence

Among eggs of the 13 *Ae. albopictus *populations transported from Madagascar to France, only six successfully hatched and gave adults for vector competence testing. The susceptibility of each population to infection was evaluated using artificial infectious CHIKV blood-meal. Overall, from a total of 1018 females tested in two trials, 1005 individuals (98.7%) were diagnosed positive as measured by IFA on head squashes (Table 7). No fluorescent signal was found in mosquitoes engorged with non-infectious blood-meal as expected. At the population level, the disseminated infection rate ranged from 94 to 100%.

**Table 7:** Disseminated infection rates of *Aedes albopictus *measured 14 days post-infection for CHIKV 06

Region	Locality	Breeding site	Total engorged mosquitoes		Total infected mosquitoes (%)	
			Trial 1*	Trial 2*	Trial1*	Trial2*
Diana	Montagne d'Ambre	Forest, tree holes	74	80	73 (98.6)	80 (100)
Atsinanana	Toamasina city	Old tires	76	77	75 (98.7)	73 (94.8)
		Coconut	90	89	89 (98.9)	89 (100)
		Buckets abandoned	96	98	94 (97.9)	97 (98.9)
Analamanga Ankazobe		Old tires	84	80	83 (98.8)	79 (98.7)
Tsimbazaza		Bamboo hedge	83	91	83 (100)	90 (98.9)

* Trial 1 was performed with generations F2 or F3, whereas trial 2 with F4 or F5

## Discussion

The survey conducted throughout two years in 15 localities among eight regions of Madagascar revealed that the distribution of *Ae. albopictus *and *Ae. aegypti *has changed considerably, in comparison to previous recorded distribution. Initially, the two species have been reported to be mainly allopatric with contrasted distribution [1,2]. Indeed, *Ae. aegypti *has been found to occupy mainly dry and semi-arid western and southern regions of Madagascar, whereas *Ae. albopictus *was dominant in eastern coast and highland areas [1,2]. Our data show that *Ae. aegypti *has become scarce within their previously delineated areas, and presently their population density is low. A decrease in *Ae. aegypti *distribution was also detected in the neighbour Reunion Island [16]. In addition, the remaining *Ae. aegypti *populations of Madagascar may have displaced to occupy several sylvatic areas: two in allopatry (Ankarana and Kirindy) and two in sympatry with *Ae. albopictus *(Montagne d'Ambre and Ranomafana). *Ae. aegypti *has been reported to occupy other forest areas on Indian Ocean Islands [2,14]. We also found two populations consisted of a few adults under bushes and fruit trees in Mahajanga city (western coast) and Diego Suarez city (north coast); which we suggest may be due to sporadic introduction by humans as this species was reported previously near the Ivato airport and in the south [2]. In contrast to its behaviour in South Asia [27], the species *Ae. aegypti *is less anthropophilic in Madagascar [1,14], consequently it is thus conceivable to hypothesize that the smaller size of such populations will provoke the extinction of the species in domestic environments.

The population density of *Ae. albopictus *was higher compared to that of *Ae. aegypti*, and also its distribution was wider, covering the eight regions surveyed from west to east and in the north. This study confirms the extension of the distribution area of *Ae. albopictus *which was already reported [2,14]), albeit to a lesser extent. More evidence hereby was provided on the adaptation capabilities of *Ae. albopictus *to occupy various eco-climatic regions of Madagascar, from the high altitude with temperate conditions prevalent in the highland region up to typical tropical conditions of the low altitude of the coastal regions. These data undoubtedly confirm the environmental plasticity of which *Ae. albopictus *is known to be capable of i.e. adaptation in contrasting environments. For instance, it was shown that *Ae*. *albopictus *has the ability to adapt to cold temperatures, and these adaptative capacities are likely due to its ability to synthesize a high amount of lipids which consequently can provide eggs with substantial yolk resources to maintain them through diapause [28-30].

*Ae. albopictus *was predominantly found in artificial environments which formed its major oviposition sites. These included dumped containers, used and abandoned tires and buckets, coconuts and bamboo cut trees, that allow for the persistence of small numbers of mosquitoes or eggs. The population density significantly increases at the rainy season, and differences were found between regions with a high abundance found in the east coast of Antsinanana. Though *Ae. albopictus *is known to be highly anthropophilic, specimens were also found in sympatry with *Ae. aegypti *in some wild areas. Surprisingly, *Ae. albopictus *also predominated in such conditions as well, suggesting a better competitiveness [31,32]. Although *Ae. albopictus *females have feeding preferences towards mammals, recent investigations highlighted the opportunistic and broad spectrum trophic behaviours to cold and other warm-blood vertebrates such as birds and reptiles [33,34]. Overall, these features suggest that the wide distribution and invasiveness of *Ae. albopictus *in Madagascar can be attributed to their adaptive behaviour to both abiotic and biotic environmental factors, including resistance to agrochemicals [35,36].

To examine whether the environment inhabited by mosquitoes generated genetic structuring amongst these populations, a phylogenetic analysis was performed using COI and ND5 mitochondrion markers [25]. The haplotype sequences of *Ae. albopictus *from Madagascar were clearly separated from those from South America (Brazil) and South Asia (Cambodia, Thailand, Vietnam). However, no significant differences were found between sylvan and domestic populations of Madagascar. Malagasy populations of *Ae. albopictus *were intermixed with individuals from the Indian Ocean (La Reunion Island), Europe (Normandie, France), and North America (Jacksonville, USA). These results may suggest a recent invasion process or a trade-off between these regions.

Experimental infections of six Malagasy *Ae. albopictus *populations with CHIKV 06.21, a strain that circulated in the Indian Ocean [8], demonstrated their high susceptibility to infection. This high susceptibility has already been reported for this CHIKV strain 06.21 in most *Ae. albopictus *populations from the Indian Ocean [9], and was attributed to an A- > V amino acid substitution at position 226 in the viral E1 glycoprotein (8), leading to efficient dissemination and transmission by the vector [9,37].

## Conclusions

Based on the current data and those obtained in previous investigations (1980s), it is clear that *Ae. albopictus *is spreading and increasing its density in Madagascar since its introduction from Southeast Asia. Concurrent with this expansion, *Ae. aegypti *has become very scarce. Changes in this biogeographical pattern are probably linked to intensive destruction of natural forests through accelerated urbanization and rapid anthropization. The increasing density of *Ae. albopictus *throughout different areas of the country and its susceptibility towards both chikungunya and dengue (5), strongly suggest that this species can be implicated as the main vector in recent outbreaks of these two arboviruses. Thus, reinforcing surveillance and implementing control measures against this invading species are needed in Madagascar. Additionally, further surveillance to monitor the biogeography of other potential mosquito vector genera such as *Culex, Mansonia*, and *Erethmapodites *which are usually associated with man-made environments is recommended.

## Competing interests

The authors declare that they have no competing interests.

## Authors' contributions

FNR managed mosquito sampling with other Malagasy members (LHR, PR, LSR). FNR, VTV, KZ, CVM, LM, ABF, PM designed and performed experiments. FNR, BOR, EH, PM

analyzed the results. FNR, BOR, PM wrote the paper in collaboration with other authors. All authors read and approved the final version of the manuscript
